# Supplementary material for: Unconventional human CD61 pairing with CD103 promotes TCR signaling and antigen-specific T cell cytotoxicity
Source: Nat Immunol. 2024 Apr 1;25(5):834–46. doi: 10.1038/s41590-024-01802-3 (PMC11065694; doi:10.1038/s41590-024-01802-3)
Supplement: Supplementary file 1 — Reporting Summary [file 41590_2024_1802_MOESM1_ESM.pdf]

nature portfolio

Corresponding author(s): Tao Dong

Last updated by author(s): Feb 15, 2024

Reporting Summary

Nature Portfolio wishes to improve the reproducibility of the work that we publish. This form provides structure for consistency and transparency in reporting. For further information on Nature Portfolio policies, see our Editorial Policies and the Editorial Policy Checklist.

Statistics

For all statistical analyses, confirm that the following items are present in the figure legend, table legend, main text, or Methods section.

n/a	Confirmed	
☐	☒	The exact sample size (*n*) for each experimental group/condition, given as a discrete number and unit of measurement
☐	☒	A statement on whether measurements were taken from distinct samples or whether the same sample was measured repeatedly
☐	☒	The statistical test(s) used AND whether they are one- or two-sided<br>*Only common tests should be described solely by name; describe more complex techniques in the Methods section.*
☐	☒	A description of all covariates tested
☐	☒	A description of any assumptions or corrections, such as tests of normality and adjustment for multiple comparisons
☐	☒	A full description of the statistical parameters including central tendency (e.g. means) or other basic estimates (e.g. regression coefficient) AND variation (e.g. standard deviation) or associated estimates of uncertainty (e.g. confidence intervals)
☐	☒	For null hypothesis testing, the test statistic (e.g. *F*, *t*, *r*) with confidence intervals, effect sizes, degrees of freedom and *P* value noted<br>*Give P values as exact values whenever suitable.*
☒	☐	For Bayesian analysis, information on the choice of priors and Markov chain Monte Carlo settings
☒	☐	For hierarchical and complex designs, identification of the appropriate level for tests and full reporting of outcomes
☐	☒	Estimates of effect sizes (e.g. Cohen's *d*, Pearson's *r*), indicating how they were calculated

Our web collection on statistics for biologists contains articles on many of the points above.

Software and code

Policy information about availability of computer code

Data collection	Flow cytometry data were collected by BD FACSDiva V9.0 on BD LSR Symphony, or Attune NxT software V3.2.1 on ThermoFisher Attune Next flow cytometer. Proteomics data were collected with either Orbitrap Fusion Lumos Tribrid platform (instrument control software v3.3) or Orbitrap Acend tribrid instrument. Miscroscopal images were collected with Olympus IX83 inverted microscope equipped with a 4-line (405 nm, 488 nm, 561 nm, and 640 nm laser) illumination system. Immunoblotting images were collected with Li-COR Odessy DLx, Li-COR Acqusition software v2.0. Immunohistochemistry images were collected using Phillips IntelliSite Pathology Solution and Visiopharm Integrator System (VIS) platform version 2020.09.0.8195.

Data analysis

Flow cytometry data were analysed with FlowJo V10.5.3 software for Mac OS, with Phenograph and tSNE plug-ins installed. Proteomics raw data were normalised to non-activated data, with STRING (https://string-db.org), NCBI Gene Ontology (https://geneontology.org) and Reactome online databases used to categorise proteins and pathways. Data was processed ion Perseus (v1.3.2.3. For ReactomePA analysis v1.32.0, Protein to gene names were converted to Entrez IDs (org.Hs.eg.db version 3.11.4) before using as input for overrepresentation analysis (clusterProfiler version 3.18.0, ReactomePA version 1.32.0) to find REACTOME pathways with enriched genes (with P value cut off 0.01 and P adjusted value cut off 0.05). The resulting output was used to create a gene concept network. Barplots were constructed for selected pathways using log2 fold change values for specific genes found in pathways (ggplot2 version 3.3.2).Volcano plots were generated using the processed data and plotted using VOlcaNOseR v1.0.3 Quantification of fluorescence intensity for microscopy experiments was performed with Fiji/ImageJ (LifeLine Java version 8) (National Institute of Health). Immunohistological analyses were done with Visiopharm v2020.09.0.8195, by first aligning adjacent slides and creating regions of overlapping positive staining to identify T cells. For survival curve Kaplan-Meier analysis, patient survival data was merged with RNA expression data for ITGB3, ITGAE, CD8A and CD3E. Optimal cutpoints were calculated to distinguish between high or low expression of each of these four genes for each dataset (survival version 3.1-12). A patient was deemed to have CD61+CD103+CD8+CD3+ cells if they showed high expression of all four genes. This was used to plot Kaplein-Meier survival curves between CD61+CD103+CD8+CD3+ patients and other remaining patients (survminer version 0.4.9, publicly-available coding).

For manuscripts utilizing custom algorithms or software that are central to the research but not yet described in published literature, software must be made available to editors and reviewers. We strongly encourage code deposition in a community repository (e.g. GitHub). See the Nature Portfolio guidelines for submitting code & software for further information.

Data

Policy information about availability of data

All manuscripts must include a data availability statement. This statement should provide the following information, where applicable:

- Accession codes, unique identifiers, or web links for publicly available datasets
- A description of any restrictions on data availability
- For clinical datasets or third party data, please ensure that the statement adheres to our policy

Raw proteomics data was deposited on Mendeley Data (dx.doi.org/10.17632/b2xdk4h5xm.1) and at the Proteome Xchange through PRoteomics IDEntifications Database (PRIDE) accession number PDX031794 and PDX045989. RNA and clinical data used for survival analysis were sourced directly from publicly available database, The Cancer Genome Atlas (TCGA) (https://portal.gdc.cancer.gov). The TCGA datasets used were the skin cutaneous melanoma (TCGA, PanCancer Atlas), lung cancer (TCGA, PanCancer Atlas) and lung cancer (University of Cologne). Patient clinical metadata for associated datasets was downloaded from cBioPortal (https://www.cbioportal.org). Raw data and reagents from all main and supplementary figures, beyond the mandatory dataset deposited on public repository, are available on request from T.D.

Human research participants

Policy information about studies involving human research participants and Sex and Gender in Research.

Reporting on sex and gender

Gender information was collected but not used for analysis in this study. The potential bias, such as the gender of patients, is unlikely to impact the final results as the samples collection and the analysis for each patient were done without any prior knowledge of this parameter, except that they are confirmed to have lung cancer but no metastasis.

Population characteristics

A total of 31 NSCLC patients were recruited and analysed for this study, ranging in age between 63 to 80 years old. Clinical parameters of patients are as informed in Extended Data Fig. 3 and Extended Data Table 2. Briefly, 7 patients are diagnosed with squamous carcinoma, 1 for plemorphic carcinoma and 23 for adenocarcinoma. 11 patients were diagnosed with TNM stage 1, 8 patients with TNM stage 2, 8 patients with TNM stage 3 and 4 patients with TNM stage 4. All patients were confirmed not to have any prior treatments and confirmed not to have metastatic cancer.

Recruitment

Confirmed non-metastatic NSCLC patients were recruited from the John Radcliffe Hospital, Oxford, United Kingdom, between December 2020 and April 2021. Subjects included both females and males who were between 63 to 80 years old. Ethics was approved by the NHS South Central – Oxford C Research Ethics Committee (REC: 19/SC/0173) under the Oxford Radcliffe Biobank (ORB) Tissue Access Committee ethics reference number 18/A026 and 20/A081. All procedures were performed according to the Declaration of Helsinki guidelines. Clinical parameters of individual patients are described in Extended Data Figure 3 and Source File 2. Patients provided voluntary informed written consent before surgery, and no compensation was provided.

Ethics oversight

Ethics oversight is carried out by the NHS South Central – Oxford C Research Ethics Committee (REC: 19/SC/0173) under the Oxford Radcliffe Biobank (ORB) Tissue Access Committee ethics reference number 18/A026 and 20/A081.

Note that full information on the approval of the study protocol must also be provided in the manuscript.

Field-specific reporting

Please select the one below that is the best fit for your research. If you are not sure, read the appropriate sections before making your selection.

☒ Life sciences ☐ Behavioural & social sciences ☐ Ecological, evolutionary & environmental sciences

For a reference copy of the document with all sections, see nature.com/documents/nr-reporting-summary-flat.pdf

Life sciences study design

All studies must disclose on these points even when the disclosure is negative.

Sample size	For the clinical cohort, a total of 31 patients with confirmed NSCLC (without prior treatment or metastasis) were included in this study. Sample sizes were based on maximal available sample sets, where detailed clinical data were also available from the biobank. For in vitro assays, each T cell assay was set up for 3 independent experiments, with sample size chosen based on the consistency between three independent replicates observed for similar functional T cell assays described previously (Peng et al., Nat Immunol, 2022, Peng et al., Nat Immuol 2020, Abd Hamid et al., Cancer Immunol Res, 2020, Abd Hamid et al., Cancer Immunol Res, 2019). For in vivo adoptive transfer model, the optimal sample size used were based on previous studies (Prota et al, Cancer Immunol Res, 2020; Shanderov et al., J Immuno Ther, 2021).
Data exclusions	For in vitro and in vivo T cells assay, the antigen-specificity are confirmed by tetramer staining prior to the assays performed, assays were carried out only if tetramer staining is at purity >90%. Additionally, T cell assays will only be carried out if the CD103 and/or CD61 expression on the T cell clones are at purity >80% (in comparison to the double negative T cell clones). For co-immunoprecipitation assay, initial flow cytometry staining of transduced primary CD8 T cells and U937 cells were performed before every assay. Co-IP will only take place if the CD103 and/or CD61 expression are at purity >80% (in comparison to the WT cells). For clinical flow cytometry analysis, no exclusion were carried out, in order to understand the full variability between patients.
Replication	For T cells mechanism and functional assays, replications of assays were performed, with 3 replicates per experiment and 3 independent repeats of the same assay, to ensure consistency between experiment of the same assay. All results gave similar successful outcome. For clinical analysis, samples were analysed on individual patients. Clinical analysis of each patients was performed once, without replication due to limited number of cells procured from tissue samples of each patient. 19 of the patients data was confirmed using two orthogonal approaches (flow cytometry and IHC), and all results gave similar successful outcome.
Randomization	Randomisation for the in vitro experiments were not relevant as input information are vital for identification of samples ran on machine such as flow cytometer. Randomisation for clinical cancer patients samples experiments were not relevant as clinical background of each patients was blinded to researcher by the Biobank before passing samples to researchers. For in vivo assay, following subcutaneous injection of tumor into the mice, at 48hours post tumor xenograft, mice were randomly allocated into two groups, with one group injected intravenously with the different T cell clones. Age-matched male and female mice were used, and randomly allocated for both groups.
Blinding	For in vivo adoptive transfer assay, T cell clones were prepared by one individual and clones were labelled with random alphabet. Allocation of mice into different groups, subcutaneous and intravenous cells injection into mice and the tumor volume measurement were carried out by another individual (from outside the main research group, ie collaborator) without prior knowledge of the association between the specific alphabet and specific T cell clones. For most clinical analysis, samples collection for each patient were done without any prior knowledge of the clinical parameters of each patient, except that they are confirmed to have lung cancer but no metastasis. Clinical parameters were referenced to and only included in the analysis when performing comparison between early stage and late stage patients.

Reporting for specific materials, systems and methods

We require information from authors about some types of materials, experimental systems and methods used in many studies. Here, indicate whether each material, system or method listed is relevant to your study. If you are not sure if a list item applies to your research, read the appropriate section before selecting a response.

Materials & experimental systems

n/a	Involved in the study	
☐	☒	Antibodies
☐	☒	Eukaryotic cell lines
☒	☐	Palaeontology and archaeology
☐	☒	Animals and other organisms
☒	☐	Clinical data
☒	☐	Dual use research of concern

Methods

n/a	Involved in the study	
☒	☐	ChIP-seq
☐	☒	Flow cytometry
☒	☐	MRI-based neuroimaging

Antibodies

Antibodies used

Flourophore	Marker	Clone ID	Supplier	Catalogue number/RRID ID	Dilution
Purified	anti-CD3/CD28	NA	StemCell Technologies	10791/RRID:AB_2827806	titer: 10ul
BUV395	anti-CD103	Ber-ACT8	BD Biosciences	564346/RRID:AB_2738759	1:33
PerCP/Cy5.5	anti-CD41	HIP8	Biolegend	303719/RRID:AB_2561732	1:33
PE	anti-CD51	NKI-M9	Biolegend	327910/RRID:AB_940564	1:33
PE/Cy7	anti-CD27	M-T271	Biolegend	256411/RRID:AB_2562258	1:33
BUV496	anti-CD28	CD28.2	BD Biosciences	741168/RRID:AB_2870741	1:33
BUV737	anti-PD-1	EH12.1	BD Biosciences	612791/RRID:AB_2870118	1:33
BB515	anti-Tim3	7D3	BD Biosciences	565568/RRID:AB_2744368	1:33
APC/Cy7	anti-CD39	A1	Biolegend	328226/RRID:AB_2571981	1:25
PerCP/Cy5.5	anti-CD45RO	UCHL1	Biolegend	304222/RRID:AB_2174124	1:33

BV650	anti-CD3	UCHT1	BD Biosciences	563851/RRID:AB_2744391	1:33
BUV805	anti-CD8	SK1	BD Biosciences	612889/ RRID:AB_2833078	1:50
BV605	anti-CD69	FN50	Biolegend	310938/RRID:AB_2562307	1:50
Purified	anti-CD103	EP206	Leica Biosystem	PA0374/NA	1:1000
Purified	anti-CD8	4B11	Leica Biosystem	PA0183/NA	1:1500
Purified	anti-CD61	VI-PL2	Abcam	AB_1086-711/NA	1:250
AF647	anti-ZAP70 (pY292)	J34-602	BD Biosciences	558515/RRID:AB_647148	1:33
AF647	anti-PLCg1 (pY783)	27/PLC	BD Biosciences	557883/RRID:AB_396921	1:25
PE	anti-Lck (pY505)	4/LCK-Y505	BD Biosciences	558552/RRID:AB_397084	1:25
AF647	anti-Vav1 pY174	EP510Y	Abcam	ab76225/RRID:AB_1524546	1:25
PE/Cy7	anti-CD107a	H4A3	Biolegend	328617/RRID:AB_11147761	1:33
BV421	anti-E-Cadherin	67A4	BD Biosciences	743712/RRID:AB_2741690	1:33
PE/Cy7	anti-CD8	SK1	Biolegend	344712/RRID:AB_2044008	1:25
Purified	anti-CD61	PM6/13	Novus Biotechnology	NBP1-28398/ RRID:AB_1853098	1:10
BV 510	anti-CD56	5.1H11	Biolegend	362534/RRID:AB_2565633	1:33
BV510	anti-CD11b	ICRF44	Biolegend	301334/RRID:AB_2562112	1:50
APC/Cy7	anti-CD3	UCHT1	BD Biosciences	557832/RRID:AB_396890	1:33
BV421	anti-CD61	VI-PL2	BD Biosciences	744381/RRID:AB_2742194	1:25
FITC	anti-CD41	HIP8	Biolegend	303704/RRID:AB_314374	1:50
FITC	anti-CD51	NKI-M9	Biolegend	327908/RRID:AB_940560	1:33
PE	anti-CD49a	T2/S7	BD Biosciences	568716/NA	1:33
BUV496	anti-CD49a	T2/S7	BD Biosciences	755215/NA	1:50
PE/Cy7	anti-CD69	FN50	Biolegend	310912/RRID:AB_314847	1:33
BV785	anti-CD39	A1	Biolegend	328240/ RRID:AB_2814191	1:25
BV421	anti-Tim-3	7D3	BD Biosciences	565562/RRID:AB_2744369	1:33
PE	anti-TIGIT	TgMab-2	BD Biosciences	568672/NA	1:33
APC/Cy7	anti-IFNg	B27	Biolegend	506524/RRID:AB_2566136	1:33
BV785	anti-TNFa	Mab11	Biolegend	502948/ RRID:AB_2565858	1:33
APC	anti-integrin beta 7	FIB504	Biolegend	321208/RRID:AB_571965	1:50
PE	anti-granulysin	DH2	Biolegend	348004/RRID:AB_2263307	1:33
AF488	anti-granzyme M	4B2G4	ThermoFisher	53-9774-42 RRID:AB_2848451	1:25
AF647	anti-granzyme B	GB11	BD Biosciences	560212/ RRID:AB_11154033	1:50
BUV737	anti-CCL5	2D7/CCR5	Biolegend	612808/RRID:AB_2870133	1:33
Purified	anti-E-Cadherin	36B5	Leica Biosystems	PA0387/NA	1:500
Purified	anti-E-Cadherin	DECMA-1	BioLegend	147302/RRID: AB_2563038	1:33
Purified	anti-CD49d	9F10	BioLegend	304302/RRID: AB_314428	1:50
Purified	anti-CD61	VI-PL2	BioLegend	336402/RRID: AB_1227584	1:33 (FC)
Purified	anti-Granzyme B	GB11	Invitrogen	MA1-80734/RRID: AB_931084	1:33
AF488	anti-CD103	Ber-ACT8	BioLegend	350208/RRID: AB_10641844	1:33
AF488	anti-TCR α/β	IP26	BioLegend	306712/RRID: AB_528967	1:33
AF647	anti-Integrin β7	FIB504	BioLegend	321222/RRID: AB_2715979	1:50
AF647	anti-CD61	VI-PL2	BioLegend	336408/RRID: AB_2128750	1:33
AF647	anti-CD103	EPR4166(2)	Abcam	ab225153/RRID:AB_2884945	1:200
Purified	anti-CD3	OKT3	Biolegend	317302/RRID:AB_571927	1:10
BV421	anti-CD103	Ber-ACT8	Biolegend	350213/RRID:AB_2563514	1:33
PE/Cy7	anti-CD61	VI-PL2	Biolegend	336416/RRID:AB_2566692	1:33
FITC	anti-integrin beta 7	FIB504	Biolegend	321212/RRID:AB_830856	1:50
AF647	anti-cMyc	9E10	Biolegend	626808/RRID:AB_2888732	1:100
PE	anti-DYKDDDDK	L5	Biolegend	637310/RRID:AB_2563148	1:100
BV421	anti-integrin beta 7	FIB504	BD Biosciences	564283/RRID:AB_2738728	1:50
PE	anti-CD61	VI-PL2	Biolegend	336406/RRID:AB_2128752	1:33
Purified	anti-FLAG	M2	Sigma-Aldrich	F3165/RRID:AB_259529	1:500
Purified	anti-b-actin	AC-74	Sigma-Aldrich	A5316/RRID:AB_476743	1:1000
Purified	anti-CD103	EPR4166(2)	Abcam	129202/RRID:AB_11142856	1:500
PE	anti-CD107a	H4A3	Biolegend	328618/RRID:AB_11147761	1:20
Purified	anti-cMyc	9E10	Sigma-Aldrich	M4439/RRID:AB_439694	1:500
BUV496	anti-CD62L	DREG-56	BD Biosciences	741155/RRID:AB_2870731	1:50
PE/Cy7	anti-CCR7	G043H7	Biolegend	353226/RRID:AB_11126145	1:50
FITC	anti-XCL2	06	Novus Biological	NBP3-06177F/NA	1:25

Validation

All antibodies used in this study are commercially available. Antibodies used in a specific species or application have been appropriately validated by manufacturers for that application and this information is provided on their website and product information datasheets. Within manufacturers website, details of verified reactivity, application used are verified by quality-testing (for flow cytometry, QC will be by flow cytometric staining of selected cell lines), with species application tested and verified are evidenced by product citations on the product webpage of the manufacturer. All antibodies described here have been further optimised for an appropriate concentration by testing several dilutions.

Eukaryotic cell lines

Policy information about cell lines and Sex and Gender in Research

Cell line source(s)

Wild-type HEK293T and HCT116 are from ATCC (CRL-3216, CCL-247). CD103 and CD61 transduced primary CD8 T cell lines

Cell line source(s)	was established in the lab. CD103+ SSX2-specific CD8 T cell clone, CD103- SSX2-specific CD8 T cell clone, CD103+ NYESO-1-specific CD8 T cell clone, CD103- NYESO-1-specific CD8 T cell clone were established in the lab.
Authentication	Cell lines from ATCC were used at the earliest passage, they were no further authentication. CD103 and CD61 transduced cell lines were verified by flow cytometry staining of CD103 and CD61.
Mycoplasma contamination	All cell lines were tested negative for mycoplasma.
Commonly misidentified lines (See ICLAC register)	No misidentified cell lines were used according to the version 11 of register of misidentified cell lines.

Animals and other research organisms

Policy information about studies involving animals; ARRIVE guidelines recommended for reporting animal research, and Sex and Gender in Research

Laboratory animals	Immunodeficient NSG mice (strain NOD.Cq-Prkdc scid Il2rgtm1Wjl/SzJ), all mice were housed in ventilated cages, maintained under specific pathogen-free conditions, with 12hrs dark/light cycle, ambient room temperature daily at 18-23 celsius, 40-60% humidity and used at 8-10 weeks old of either sex.
Wild animals	The study did not involve wild animals.
Reporting on sex	Sex information was not used in the analysis of the in vivo model, because the model used are based on SCID mice system and the human tumor xenograft (that express the specific HLA and tumor antigen) recognised specifically by the human T cells used for adoptive transfer. Mice hormonal and its immune system would not affect the outcome of the data, because the cancer cell killing will be directed specifically by human TCR-pMHC interaction (plus action of co-stimulatory/inhibitory receptors). Therefore, age-matched male and female mice were used for both treatment groups..
Field-collected samples	The study did not involves samples collected from the field.
Ethics oversight	All mice experiments were performed in accordance with Animals (Scientific Procedures) Act 1986 and according to the University of Oxford Animal Welfare and Ethical Review Body (AWERB) guidelines, and operating under the UK Home Office license PBA43A2E4.

Note that full information on the approval of the study protocol must also be provided in the manuscript.

Flow Cytometry

Plots

Confirm that:

☒ The axis labels state the marker and fluorochrome used (e.g. CD4-FITC).

☒ The axis scales are clearly visible. Include numbers along axes only for bottom left plot of group (a 'group' is an analysis of identical markers).

☒ All plots are contour plots with outliers or pseudocolor plots.

☒ A numerical value for number of cells or percentage (with statistics) is provided.

Methodology

Sample preparation	For ex vivo samples, For the ex vivo multicolour flow cytometry, cell suspension used for the assay was isolated from tissue as previously described20. Briefly, tissues were cut into small pieces using a pair of scissor and forceps before enzymatically dissociated into cell suspensions in RPMI-1640 using human tumor dissociation kit (Miltenyi Biotech), following protocol provided by the supplier. Following the enzymatic dissociation, cells were filtered through 100um strainer to remove indigestible parts of the tissue, with dead cells or debris were removed by centrifugation at 1,500 rpm for 10 minutes. Cells were then resuspended in RPMI-1640 supplemented with 10% FCS (Sigma Aldrich), 2 mM L-glutamine (Sigma Aldrich) and 1% v/v (500U/ml) penicillin/streptomycin (Sigma Aldrich). For peripheral blood, the PBMC were isolated using Ficoll-Hypaque gradient isolation, followed by centrifugation to pellet the PBMC and resuspended in the same complete media as above.For each immunophenotyping staining, 1M cells of paratumor tissue, tumor and peripheral blood were first stained with Live/Dead Fixable Aqua Stain Kit (ThermoFisher) for 20 minutes at 4C. For surface staining, cells were washed and then stained with dumping markers: BV510 anti-CD56 (Biolegend) and BV510 anti-CD11b (Biolegend), T cells markers: BUV805 anti-CD8 (BD Biosciences) and BV650 or APC/Cy7 anti-CD3 (BD Biosciences), integrins: BUV395 anti-CD103 (BD Biosciences), BV421 or AF647 anti-CD61 (BD Biosciences), PerCP/Cy5.5 or FITC anti-CD41 (Biolegend), APC anti- integrin B7 (Biolegend) and PE or FITC anti-CD51 (Biolegend), tissue-resident T cell markers: PerCP/Cy5.5 anti-CD45RO (Biolegend), PE or BUV496 anti-CD49a (BD Biosciences) and PE/Cy7 or BV605 anti-CD69 (Biolegend), tumor-reactive TILs marker: APC/Cy7 or BV785 anti-CD39 (BD Biosciences and Biolegend), T cell differentiation markers: PE/Cy7 anti-CD27 (BD Biosciences) and BUV496 anti-CD28 (BD Biosciences), inhibitory markers: BUV737 anti-PD-1 (BD Biosciences), BV421 or BB515 anti-Tim-3 (BD Biosciences) and PE anti-TIGIT (BD Biosciences) for another 20 minutes at 4C. For intracellular cytokine staining, cells were T cells were treated with 0.7ug/ml Monensin and 1ug/ml Brefeldin A (BD Biosciences), washed and permeabilised with BD CytoFix/CytoPerm Solution for 20 minutes at 4C, before stained with cytokines: APC/Cy7 anti-IFNg (Biolegend) and BV785 anti-TNFa (Biolegend), cytolytic molecules: PE anti-granulysin (Biolegend), AF488 anti-granzyme M (ThermoFisher), AF647 anti-granzyme B (BD Biosciences), chemokines: BUV737 anti-CCL5 (Biolegend) and FITC anti-XCL2 for another 20 minutes at 4C. Following antibodies staining, cells were fixed with 1X CellFix (BD Biosciences) and acquired on BD LSR Symphony (BD

Biosciences) and analysed on FlowJo V.10 (BD Biosciences).
For in vitro assays, T cell lines and clones were T cells were co-culture with cancer cells at an E:T ratio of 1:10 at 37C for 15 minutes, 30 minutes or 2 hours. Following T cells activation, cells were stained with Live/Dead Fixable Aqua Dead Cell Stain Kit (Thermo Fisher) before fixed with BD Cytofix Fixation Buffer for 10 minutes at 37C. Cells were then permeabilised using BD Phosflow Perm Buffer III for 30 minutes at 4C before stained with FITC anti-ZAP70 (BD Biosciences), AF647 anti-ZAP70 (pY292) (BD Biosciences), AF488 anti-PCLg1 (BD Biosciences), AF647 anti-PLCg1 (pY783) (BD Biosciences), PE anti-Lck (pY505) (BD Biosciences), AF647 anti-Lck (BD Biosciences) and AF647 anti-Vav1 (pY174) (Abcam). Cells were then acquired immediately on Attune Nxt flow cytometer (ThermoFisher) and analysed on FlowJo V.10 (BD Biosciences). To assess contribution of CD61 towards TCR signalling proteins activities, T cells were treated with 10nM aminogenistein (Lck inhibitor, Santa Cruz Biotechnology) before the T cells activation and the Phosflow assay were performed (Phosflow assay). For T cell cytotoxicity assay, Cancer cells were initially stained with 0.5ug/ml CFSE (ThermoFisher) prior to co-culture with T cells at an E:T ratio of 1:10 at 37C for either 2, 4, 6 and 8 hours. Cells were then stained with 7-AAD (BD Biosciences) and BV421 anti-E-Cadherin (Biolegend) and PE/Cy7 anti-CD8 (BD Biosciences) before acquiring on the Attune Nxt flow cytometer (ThermoFisher) and analysed on FlowJo V.10 (BD Biosciences). To evaluate the T cell cytotoxic efficacy, the WT T cells were treated with anti-CD61 (10ug/ml, PM6/13, Novus Biotechnology), in parallel to the T cells activation. For T cell proliferation assay, 1M cells of paratumor tissue or of tumor tissue were stained with 0.5ug/ml CFSE prior to activation with 10ul anti-CD3/CD28 (StemCell Technologies). The cells were incubated at 37C for 72 hours. After, the cells were stained with Live/Dead Fixable Aqua Cell Stain Kit (Thermo Fisher) for 20 minutes at 4C before being stained with conjugated antibodies against BV650 anti-CD3 (BD Biosciences), BUV805 anti-CD8 (BD Biosciences), BUV395 anti-CD103 (BD Biosciences), BV421 anti-CD61 (BD Biosciences), PerCP/Cy5.5 anti-CD45RO (Biolegend), PE anti-CD49a (BD Biosciences and Biolegend) and PE/Cy7 anti-CD69 (Biolegend). Following antibodies staining, cells were fixed with 1X CellFix (BD Biosciences) and acquired on BD LSR Symphony (BD Biosciences) and analysed on FlowJo V.10 (TreeStar Inc.). Cells were considered proliferative based on decrease in CFSE fluorescence, within the 1st downward peaks of CFSE onwards.

Instrument

Samples were acquired on BD LSR Symphony or ThermoFisher Attune Nxt Flow Cytometer.

Software

Data were collected using FACS Diva v9.0.1 or Attune NXt software V3.2.1 and analysed using FlowJo v10 software for Mac OS.

Cell population abundance

For the clinical samples, cells were not sorted, all analysis were done with multicolour flow cytometry. The specific population of interest ex vivo ranges from 2% to 78%. For the cancer-specific T cell clones, the process of identification, processing, sorting and in vitro culture is as previously described (Abd Hamid et al., Cancer Immunol Res, 2020). CD103 and/or CD61 transduced cell lines were not sorted before performing co-IP, as the purity is high (>80%).

Gating strategy

For all flow cytometry-based experiments, cells were first gated on single lymphocytes by a forward side scatter gate. Identification of live T cells were done based on excluding dead cells (by Fixable Live/Dead staining), CD3 and CD8 staining, before identification for CD103+ CD61+ cells. For intracellular cytokine staining (ICS), phosflow staining and integrins validation on transduced U937 cell lines, positive/negative population were gated according to corresponding negative controls, either unstimulated samples or wild-type unstimulated/non-transduced samples.

☒ Tick this box to confirm that a figure exemplifying the gating strategy is provided in the Supplementary Information.
