# Supplementary material for: Unconventional human CD61 pairing with CD103 promotes TCR signaling and antigen-specific T cell cytotoxicity
Source: Nat Immunol. 2024 Apr 1;25(5):834–46. doi: 10.1038/s41590-024-01802-3 (PMC11065694; doi:10.1038/s41590-024-01802-3)
Supplement: Supplementary file 5 — Unprocessed western blot. [file 41590_2024_1802_MOESM5_ESM.pdf]

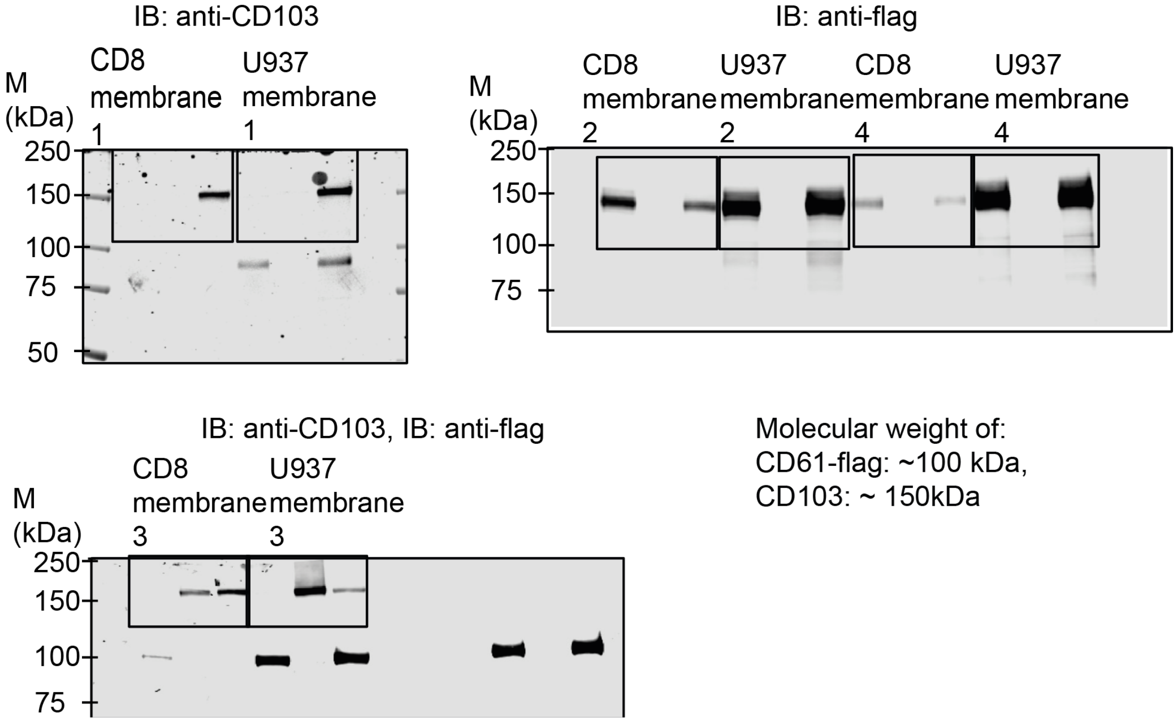

Source File 3. CD61 interacts with CD103. Full immunoblot images for CD103 and CD61-flag on whole cell lysate (WCL) and pulldown lysates, of integrins-transduced primary T cell and U937 cells. Immunoblot of CD103 on pulldown lysate of transduced T cell (CD8 membrane 1), of transduced U937 (U937 membrane 1) (*top*). Immunoblot of CD61-flag on pulldown lysate of transduced T cell (CD8 membrane 2), of transduced U937 (U937 membrane 2), on WCL of transduced T cell (CD8 membrane 4), of transduced U937 (U937 membrane 4) (*middle*). Immunoblot of CD103 and CD61-flag on WCL of transduced T cell (CD8 membrane 3), of transduced U937 (U937 membrane 3) (*bottom*). Molecular weight of CD61-flag: ~100 kDa, of CD103: ~150kDa. U937 is used as a positive control of transduction.
